# Healthy Lifestyle Behaviors Associated with Subjective Well-Being at School in Adolescents

**DOI:** 10.3390/healthcare14142151

**Published:** 2026-07-16

**Authors:** Hüseyin Selvi, Aydolu Algın Toros

**Affiliations:** 1 Department of Medical Education, Faculty of Medicine, Mersin University, Mersin 33343, Türkiye; 2 Department of Nursing, Faculty of Health Sciences, Burdur Mehmet Akif Ersoy University, Burdur 15200, Türkiye; aydolualgin@hotmail.com

**Keywords:** adolescents, nutrition behavior, exercise behavior, subjective well-being at school, school satisfaction

## Abstract

**Highlights:**

**What are the main findings?**

- Healthy nutrition and exercise behaviors were positively associated with subjective well-being at school.
- Psychological/dependent eating was negatively associated with subjective well-being at school.

**What are the implications of the main findings?**

- School-based lifestyle interventions may help support adolescents’ subjective well-being at school.
- Promoting healthy lifestyle behaviors during adolescence may help inform long-term health promotion strategies.

**Abstract:**

**Background/Objectives**: This study aimed to examine whether regular exercise and healthy nutrition behaviors are associated with subjective well-being at school among adolescents. **Methods**: This descriptive study was conducted with 272 high school students aged 15–18 years who participated on a voluntary basis. Data were collected using the Nutrition and Exercise Behavior Scale, the Subjective Well-Being at School Scale, and a demographic information form. Descriptive statistics, group comparisons, correlation analyses, and regression analyses were performed to evaluate the relationships between lifestyle behaviors and subjective well-being at school. **Results**: The mean age of the participants was 16.27 ± 0.88 years; 55.9% were female and 44.1% were male. According to Body Mass Index (BMI) classification, 15.4% were underweight, 62.9% were normal weight, 16.2% were overweight, and 5.5% were obese. No statistically significant differences were found in subjective well-being at school scores according to gender or BMI categories (*p* > 0.05). Regression analysis indicated that healthy nutrition and exercise behaviors were positively associated with subjective well-being at school (β = 0.380, *p* < 0.001), whereas psychological/dependent eating behaviors were negatively associated with subjective well-being at school (β = −0.171, *p* = 0.043). **Conclusions**: Healthy nutrition and exercise behaviors were significantly associated with subjective well-being at school among adolescents. School-based lifestyle interventions may help support adolescents’ well-being at school and may inform future health promotion strategies.

## 1. Introduction

Regular exercise and healthy nutrition are fundamental lifestyle factors that influence not only individuals’ physical health but also their psychological and social well-being [1,2]. Adolescence is a critical developmental period characterized by rapid physical, emotional, and social changes, during which identity formation takes place and lifelong health behaviors and lifestyle patterns are established. Behaviors acquired during this stage play a significant role in shaping individuals’ future health status, psychological adjustment, and overall quality of life [3,4].

Adolescence is also a period in which individuals increasingly interact with their social environment, develop stronger peer relationships, and construct their social identity. During this process, social well-being is closely associated with factors such as social integration, sense of belonging, perceived social support, and the quality of interpersonal relationships. Therefore, lifestyle behaviors developed during adolescence may have important implications not only for physical and psychological health but also for social well-being [5].

Previous studies have shown that regular physical activity plays an important role in preventing obesity and overweight while improving overall health status [1,2]. Moreover, physical activity contributes to psychological well-being by enhancing self-esteem, self-efficacy, and social skills, and by promoting active participation in social life [2,6].

Furthermore, physical activity has been shown to increase interpersonal interactions, strengthen social bonds, and support a sense of community [7]. Adolescents who engage in regular exercise tend to develop stronger social relationships [8], participate more actively in social environments, and tend to report higher levels of social adjustment. In this respect, physical activity is considered an important factor not only for individual health but also for supporting social well-being. However, some studies have reported inconsistent findings regarding the relationships between lifestyle behaviors and adolescent well-being, suggesting that these associations may vary depending on psychosocial, environmental, and contextual factors.

Similarly, healthy dietary habits contribute to maintaining physical health by ensuring adequate and balanced nutrient intake and reducing the risk of obesity and related health problems [9]. Increasing evidence has demonstrated a strong relationship between nutrition and mental health [9,10].

Healthy eating habits have been associated with lower levels of depression and anxiety, improved stress regulation, and enhanced cognitive functioning [11]. Moreover, dietary behaviors may influence social behaviors and decision-making processes. In contrast, irregular and psychologically driven eating patterns may negatively affect emotional regulation and, consequently, impair social adjustment [12]. Social well-being is defined as a multidimensional construct that encompasses individuals’ functioning within society, the quality of their social relationships, and their interactions with the social environment [13]. In the context of school, social well-being includes components such as school satisfaction, positive affect, social acceptance, and peer relationships [14]. Therefore, subjective well-being at school is considered an important indicator of adolescents’ overall life satisfaction and psychosocial adjustment. In the present study, subjective well-being at school was considered a context-specific indicator of adolescents’ psychosocial and social well-being within the school environment.

Although the present study was not designed within a specific theoretical framework, the findings may be broadly interpreted within Social-Ecological perspectives emphasizing that adolescent well-being is shaped by the interaction of behavioral, individual, and environmental factors [15].

Examining physical activity and healthy nutrition behaviors together may provide a more comprehensive understanding of their role in supporting social well-being among adolescents.

However, existing studies have generally examined physical activity, dietary behaviors, and subjective well-being separately, and evidence addressing their combined associations with subjective well-being at school among adolescents remains limited [16]. This highlights the need for more comprehensive and integrative research in this field. In addition, relatively few studies have examined healthy and unhealthy nutrition–exercise behaviors together within the context of adolescents’ subjective well-being at school.

Understanding these relationships may provide important implications for developing school-based interventions aimed at improving adolescents’ well-being.

Therefore, the present study aims to examine the relationship between regular exercise, healthy nutrition behaviors, and subjective well-being at school among adolescents. Specifically, this study addresses the following research questions:

1. What are the levels of regular exercise, healthy nutrition behaviors, and subjective well-being at school among adolescents?
2. Is there a significant relationship between healthy nutrition/exercise behaviors and subjective well-being at school among adolescents?
3. Are healthy nutrition and exercise behaviors significantly related to subjective well-being at school?

## 2. Materials and Methods

This study was designed as a cross-sectional descriptive study [17].

### 2.1. Participants

The study population consisted of adolescents attending various high schools in Mersin, Türkiye. Participants were recruited using a convenience sampling approach based on voluntary participation. Students who agreed to participate and met the inclusion criteria were included in the study.

A priori power analysis was conducted using G*Power version 3.1.9.7 based on a linear multiple regression model (fixed model, R^2^ deviation from zero). The analysis included six predictors and was performed using a small effect size (f^2^ = 0.10), an alpha level of 0.05, and a statistical power of 0.80. A small effect size was selected considering the exploratory nature of the study and the multifactorial nature of psychosocial variables in adolescent populations. The minimum required sample size was calculated as 120 participants. In total, data were obtained from 272 students who met the inclusion criteria.

The inclusion criteria were:

- Being between 15 and 18 years of age;
- Being enrolled in a high school in Mersin;
- Providing voluntary consent to participate.

The exclusion criteria included:

- Being outside the specified age range;
- Having a diagnosed chronic disease or psychiatric disorder.

Participants and their parents/guardians were informed about the purpose and procedures of the study. Participation was voluntary, and informed consent and assent were obtained prior to data collection. During data checking, two implausible age entries were identified and treated as likely data-entry errors. Because the remaining questionnaire responses from these participants were complete, only the age values were treated as missing in age-related analyses, while the participants remained in the dataset.

The study was conducted in accordance with the Declaration of Helsinki and approved by Mersin University Ethics Committee (Decision No: 52, 4 March 2025). Informed consent was obtained from participants.

### 2.2. Data Collection Instruments

Data were collected using the Nutrition and Exercise Behavior Scale, the Subjective Well-Being at School Scale, and a demographic information form. Permission for the use of the scales was obtained from the original authors.

### 2.3. Data Collection Procedure

Data collection was conducted in collaboration with high schools in Mersin, Türkiye. Participants were informed about the purpose and voluntary nature of the study before participation.

The questionnaires were administered both online and in paper-based format. Most participants completed the questionnaires online via Google Forms during school hours in classroom settings under teacher supervision. Some students completed printed paper forms in the classroom environment, while others completed the online forms at home under parental supervision.

All participants completed the questionnaires individually and voluntarily. Efforts were made to ensure similar administration conditions throughout the data collection process.

Data collection was conducted in April and May 2025.

### 2.4. Nutrition and Exercise Behavior Scale (NEBS)

The Nutrition and Exercise Behavior Scale, developed by Yurt and Save, consists of 45 items and four subscales and is scored on a five-point Likert scale. The scale assesses individuals’ nutrition and exercise behaviors [18].

The subscales include:

Psychological/Dependent Eating Behavior (11–55 points);
Healthy Nutrition–Exercise Behavior (14–70 points);
Unhealthy Nutrition–Exercise Behavior (14–70 points);
Meal Regularity (6–30 points).

Higher scores indicate stronger tendencies in the corresponding behavioral domain. The scale does not have a defined cut-off score. The scale has previously demonstrated acceptable psychometric properties, including validity and reliability, in adolescent samples.

### 2.5. Subjective Well-Being at School Scale

The Subjective Well-Being at School Scale is a 10-item Likert-type instrument developed to assess students’ subjective well-being in the school context, including both cognitive and affective components. The scale is rated on a six-point Likert scale. The School Satisfaction subscale consists of 8 items (score range: 8–48), and the School-related Emotions subscale consists of 2 items (score range: 2–12). The Turkish adaptation was conducted by Özdemir and Sağkal [19]. Previous findings from the Turkish adaptation study demonstrated satisfactory psychometric properties, with Cronbach’s alpha coefficients reported as 0.88 for school satisfaction and 0.76 for the affective dimension [19]. No cut-off score has been defined for this instrument.

### 2.6. Demographic Information Form

A four-item questionnaire developed by the researchers was used to obtain participants’ demographic characteristics, including age, gender, grade level, and sports participation.

Height and weight values were self-reported. Body Mass Index (BMI) classifications were calculated according to age and sex-specific percentile values (<5th percentile underweight, 5th–85th percentile normal weight, 85th–95th percentile overweight, and ≥95th percentile obese).

### 2.7. Reliability Analysis

The reliability of the scales used in the study was evaluated using Cronbach’s alpha and McDonald’s omega coefficients. In the present study, Cronbach’s alpha was 0.84 and McDonald’s omega was 0.85 for the Subjective Well-Being at School Scale. For the Nutrition and Exercise Behavior Scale, Cronbach’s alpha was 0.89 and McDonald’s omega was 0.89, indicating high internal consistency for both scales.

Cronbach’s alpha and McDonald’s omega coefficients for the subscales were as follows: School Satisfaction (α = 0.84, ω = 0.85), School-related Emotions (α = 0.60, ω = 0.60), Psychological/Dependent Eating Behavior (α = 0.69, ω = 0.70), Healthy Nutrition–Exercise Behavior (α = 0.74, ω = 0.75), Unhealthy Nutrition–Exercise Behavior (α = 0.68, ω = 0.69), and Meal Regularity (α = 0.80, ω = 0.80). The relatively low reliability coefficient observed for the School-related Emotions subscale may partly be attributable to the limited number of items in this dimension. In addition, the internal consistency coefficients for some Nutrition and Exercise Behavior Scale subscales were slightly below the conventional 0.70 threshold and should therefore be interpreted cautiously.

### 2.8. Statistical Analysis

Data were analyzed using Statistica^®^ 13.5.0.17. Descriptive statistics were presented as frequency (*n*), percentage (%), mean ± standard deviation, median, and interquartile range, depending on the distribution of the variables.

Normality of continuous variables was assessed using the Shapiro–Wilk test. For normally distributed variables, parametric tests were applied, whereas non-parametric tests were used when normality assumptions were not met.

Comparisons between two independent groups were conducted using the Independent Samples *t*-test or Mann–Whitney U test, while comparisons among three or more groups were performed using One-Way ANOVA or the Kruskal–Wallis H test.

Because no group comparison reached the uncorrected α = 0.05 threshold, multiple-comparison correction did not alter the inferential conclusions. Benjamini–Hochberg false discovery rate (FDR)-adjusted *p*-values were calculated and are reported for transparency. Relationships between variables were examined using the Spearman correlation coefficient. Statistical significance was set at *p* < 0.05.

Multiple linear regression analysis was performed to examine the associations between subjective well-being at school (dependent variable) and age, BMI, and Nutrition and Exercise Behavior Scale subscale scores (independent variables). Prior to regression analysis, assumptions related to multicollinearity and approximate normality of residual distributions were evaluated. Multicollinearity diagnostics were assessed using tolerance and Variance Inflation Factor (VIF) values, and all values were within acceptable limits. Multiple linear regression analysis was performed to examine the associations between subjective well-being at school (dependent variable) and age, BMI, and Nutrition and Exercise Behavior Scale subscale scores (independent variables). Prior to regression analysis, assumptions related to multicollinearity and approximate normality of variable distributions were evaluated. Multicollinearity diagnostics were assessed using tolerance and Variance Inflation Factor (VIF) values, and all values were within acceptable limits. In addition, regression assumptions were further evaluated through examination of standardized residuals, Q-Q plots, Shapiro–Wilk test of residual normality, and Cook’s distance values for influential observations.

## 3. Results

A total of 272 adolescents aged 15–18 years who were attending high schools in Mersin and met the inclusion criteria participated in the study. The mean age of the participants was 16.27 ± 0.88 years. The mean body weight and height were 64.10 ± 15.40 kg and 168.3 ± 10.8 cm, respectively. Of the participants, 55.9% (*n* = 152) were female and 44.1% (*n* = 120) were male. Based on BMI classification, 15.4% were underweight, 62.9% were normal weight, 16.2% were overweight, and 5.5% were obese. Detailed demographic characteristics are presented in Table 1.

### 3.1. Comparison by Gender

Differences in subjective well-being at school and nutrition–exercise behaviors according to gender were examined using the Mann–Whitney U test. No statistically significant differences were found between male and female participants across all variables, including school satisfaction, School-related emotions, psychological/dependent eating behavior, healthy nutrition–exercise behavior, unhealthy nutrition–exercise behavior, and meal regularity (*p* > 0.05). Gender-based comparisons are presented in Table 2.

### 3.2. Comparison by BMI Categories

Group comparisons based on BMI categories were conducted using the Kruskal–Wallis H test. No statistically significant differences were observed among BMI groups in terms of subjective well-being at school or nutrition–exercise behavior subscale scores (*p* > 0.05). The results of BMI-based comparisons are shown in Table 3.

### 3.3. Correlation Analysis

Relationships between the study variables were examined using Spearman correlation analysis. Subjective well-being at school showed weak positive correlations with healthy nutrition–exercise behavior (r = 0.270 for school satisfaction and r = 0.192 for School-related emotions, *p* < 0.01). Smaller positive correlations were also observed between unhealthy nutrition–exercise behavior and school satisfaction (r = 0.123, *p* < 0.05) as well as School-related emotions (r = 0.146, *p* < 0.05). No significant correlations were found between subjective well-being and psychological/dependent eating behavior or meal regularity. Significant positive correlations were also observed among several subscales of the Nutrition and Exercise Behavior Scale. The full correlation matrix is presented in Table 4.

### 3.4. Regression Analysis

A multiple linear regression analysis was conducted to examine the associations between subjective well-being at school and age, BMI, and the subscales of the Nutrition and Exercise Behavior Scale. The regression model was statistically significant (F(6, 265) = 4.769, *p* < 0.001) and explained 9.7% of the variance in subjective well-being at school (R^2^ = 0.10, adjusted R^2^ = 0.08). The effect size of the model was small (Cohen’s f^2^ = 0.11). Healthy nutrition–exercise behavior was significantly and positively associated with subjective well-being at school (B = 0.527, β = 0.380, *p* < 0.001; 95% CI [0.23, 0.63]), whereas psychological/dependent eating behavior was significantly and negatively associated with subjective well-being (B = −0.239, β = −0.171, *p* = 0.043; 95% CI [−0.47, −0.01]). Age, BMI, unhealthy nutrition–exercise behavior, and meal regularity were not significantly associated with subjective well-being at school (*p* > 0.05). Collinearity diagnostics indicated no serious multicollinearity problems among the predictor variables (tolerance = 0.395–0.974; VIF = 1.027–2.533). Detailed regression results are presented in Table 5.

Examination of regression assumptions indicated no major violations. The standardized residuals were approximately normally distributed (Shapiro–Wilk = 0.989, *p* = 0.072), visual inspection of residual and Q-Q plots did not indicate substantial deviations from normality or homoscedasticity, and Cook’s distance values did not suggest influential outliers (maximum Cook’s distance = 0.697).

In addition, Harman’s single-factor test was conducted to assess the potential impact of common method bias. The un-rotated single-factor solution explained 14.74% of the total variance, which was substantially below the recommended 50% threshold, suggesting that common method bias was unlikely to seriously affect the findings of the study.

## 4. Discussion

The present study investigated whether regular exercise and healthy nutrition behaviors are associated with subjective well-being at school among adolescents. The findings suggest that healthy lifestyle behaviors are associated with adolescents’ subjective well-being at school and support the growing body of literature emphasizing the multidimensional associations between healthy lifestyle habits and psychological and social functioning.

The findings indicated that healthy nutrition and exercise behaviors were significantly associated with subjective well-being at school. In particular, higher levels of healthy nutrition and exercise behaviors were associated with higher levels of subjective well-being at school, whereas psychological/dependent eating behaviors were negatively associated with well-being. These findings are consistent with previous studies reporting that regular physical activity and healthy lifestyle behaviors are associated with better subjective well-being at school and lower levels of depression, anger, and stress [20]. Physical activity has been reported to promote not only physical health but also important components of subjective well-being at school such as social interaction, a sense of belonging, and self-efficacy [20]. Previous studies has also shown that individuals who exercise regularly, particularly two to three times per week, report stronger feelings of social integration and more positive interpersonal relationships [21]. Similarly, adolescents who engage in regular physical activity tend to report higher self-esteem, stronger peer relationships, and lower levels of depressive symptoms.

Healthy eating habits may also be related to adolescents’ psychological and social functioning. Evidence suggests that balanced dietary patterns can support emotional regulation, reduce stress reactivity, and influence social decision-making processes [11]. In the present study, healthy nutrition–exercise behaviors and meal regularity were positively correlated with subjective well-being at school, whereas psychological/dependent eating behaviors were negatively associated with subjective well-being at school. These findings highlight the potential role of lifestyle behaviors in shaping adolescents’ emotional and social adjustment.

Although weak positive correlations were also observed between unhealthy nutrition–exercise behaviors and subjective well-being dimensions, these associations were considerably weaker than those observed for healthy nutrition–exercise behaviors and should therefore be interpreted cautiously. These findings may reflect the complex and context-dependent nature of adolescent lifestyle behaviors and peer-related social interactions [22].

Another important finding of the study was that no significant differences in subjective well-being at school were observed according to gender or BMI categories. This result suggests that adolescents’ subjective well-being at school may be influenced more strongly by behavioral, psychosocial, and environmental factors than by demographic or physical characteristics alone. Previous research has similarly indicated that adolescent well-being is shaped by a complex interaction of lifestyle behaviors, environmental influences, and social relationships rather than by biological characteristics alone [21].

However, some findings of the present study differ from previous research reporting gender and BMI-related differences in psychological well-being and lifestyle behaviors. These inconsistencies may be associated with cultural characteristics, differences in sample composition, variations in measurement tools, or contextual factors related to the school environment. In addition, the causal mechanisms underlying the relationships between lifestyle behaviors and subjective well-being remain unclear [9]. Therefore, the inconsistent findings across studies further emphasize the complex and multidimensional nature of adolescent well-being.

The regression analysis further supported these findings by indicating that subjective well-being at school was more strongly associated with behavioral and psychological factors than with demographic variables such as age or BMI. In particular, psychological/dependent eating behavior was negatively associated with subjective well-being, which is consistent with previous studies reporting that maladaptive eating patterns are associated with emotional regulation difficulties and psychosocial adjustment problems in adolescents [23]. In contrast, healthy nutrition and exercise behaviors were positively associated with subjective well-being at school, supporting previous evidence suggesting that healthy lifestyle behaviors are related to better psychological adjustment and higher school satisfaction among adolescents [6,24]. The pattern in which unhealthy nutrition–exercise behavior demonstrated a weak but significant zero-order correlation with subjective well-being but a non-significant regression coefficient in the multivariate model may reflect a suppression effect arising from shared variance among lifestyle-related predictors. Therefore, the weak positive bivariate associations observed for unhealthy nutrition–exercise behaviors should be interpreted cautiously rather than as stable independent relationships. The relatively small effect size observed in the present study (f^2^ = 0.11) is also generally consistent with previous literature suggesting that adolescent well-being is influenced by multiple psychosocial and contextual factors beyond lifestyle-related behaviors alone.

Although the regression model was statistically significant, the explained variance was relatively low (R^2^ = 0.10), indicating that the variables included in the model accounted for only a limited proportion of the variability in subjective well-being at school. This finding suggests that subjective well-being at school is a complex and multidimensional construct likely influenced by numerous psychosocial, environmental, and contextual factors beyond nutrition and exercise behaviors alone. Variables such as family environment, peer relationships, socioeconomic conditions, psychological characteristics, school climate, and perceived social support may also play important roles in adolescents’ subjective well-being at school. In addition, the effect size of the regression model was small (Cohen’s f^2^ = 0.11), further supporting the need for cautious interpretation of the findings.

It should also be noted that some variables that were not significantly associated with subjective well-being in the bivariate correlation analysis became significant in the multivariate regression model. This finding may be explained by the shared variance and interrelationships among variables included in the regression model. In multivariate models, controlling for other related variables may reveal associations that are not evident in bivariate analyses alone. Therefore, the regression findings should be interpreted cautiously within the context of the overall multivariate structure of the model. Future studies including broader psychosocial and environmental variables may provide a more comprehensive understanding of the factors associated with adolescent well-being.

The findings of the present study may have important implications for school-based health promotion programs aimed at improving adolescents’ subjective well-being at school. Interventions supporting healthy nutrition and regular physical activity may be associated with better physical health and subjective well-being among adolescents.

Future research should employ longitudinal and experimental designs to better clarify the directionality and causal mechanisms underlying the relationships between lifestyle behaviors and adolescent well-being. In addition, future studies including broader psychosocial, familial, and environmental variables may provide a more comprehensive understanding of the factors associated with adolescents’ subjective well-being.

### 4.1. Theoretical Implications

The findings of the present study contribute to the growing literature emphasizing the multidimensional nature of adolescent subjective well-being at school. The results suggest that lifestyle-related behaviors may be associated with adolescents’ psychosocial adjustment within school settings, although these relationships appear to be relatively modest and influenced by multiple behavioral and contextual factors. In particular, the findings support the view that healthy nutrition and exercise behaviors should be evaluated within broader psychosocial and environmental contexts when examining adolescent well-being.

### 4.2. Limitations

The findings of the present study should be interpreted in light of several limitations.

First, the cross-sectional design limits the ability to establish causal relationships between healthy lifestyle behaviors and subjective well-being at school. Therefore, the observed relationships should be interpreted as associative rather than causal. Longitudinal and experimental studies would provide a stronger methodological basis for clarifying the directionality and underlying mechanisms of these associations.

Second, the study relied on self-reported data, including height and weight measurements used for BMI calculation. Since adolescents may underreport their weight or overreport their height, measurement bias may have occurred, potentially affecting the accuracy of BMI classifications and related analyses. In addition, because all study variables were collected using self-report measures, common method bias cannot be completely ruled out, although Harman’s single-factor test suggested that common method variance was unlikely to substantially affect the findings.

Third, the use of a convenience sampling method based on voluntary participation may have introduced selection bias, as adolescents who are more interested in health-related behaviors may have been more likely to participate in the study.

Finally, since the sample consisted only of high school students from a single geographical region, the generalizability of the findings to broader adolescent populations may be limited.

In addition, subjective well-being at school is a complex and multidimensional construct that may also be influenced by several psychosocial and environmental factors, such as family environment, peer relationships, socioeconomic status, school climate, perceived social support, sleep patterns, and screen time, which were not included in the present study.

Furthermore, measurement invariance of the Subjective Well-Being at School Scale across demographic groups was not examined in the present sample. In addition, the internal consistency coefficients for some subscales were relatively low or slightly below the conventional 0.70 threshold. In particular, the School-related Emotions subscale consisted of only two items, which may have contributed to the relatively low reliability coefficients observed for this dimension.

## 5. Conclusions

Overall, the findings of this study highlight the potential importance of promoting healthy lifestyle behaviors during adolescence. School environments may play an important role in encouraging healthy eating habits and regular physical activity, which may in turn be associated with adolescents’ subjective well-being at school and psychological adjustment.

The results of this study suggest that regular exercise and healthy nutrition are important behavioral factors associated with subjective well-being at school among adolescents. Promoting healthy lifestyle behaviors in school settings may be associated with better social integration, self-esteem, and psychological adjustment among young people. Given the multidimensional nature of subjective well-being at school, interventions aimed at improving adolescents’ well-being should consider not only physical health behaviors but also psychosocial and environmental factors.

However, due to the cross-sectional nature of the study and the relatively low explained variance of the regression model, the findings should be interpreted cautiously and causal relationships cannot be inferred. Future longitudinal and intervention-based studies are needed to better understand the complex relationships between lifestyle behaviors and subjective well-being at school, including the potential mediating roles of family, peers, social support systems, and school environments.

## Figures and Tables

**Table 1:** Demographic characteristics of participants.

Variable		Value
Age (years), mean ± SD		16.2 ± 0.8
Weight (kg), mean ± SD		64.1 ± 15.4
Height (cm), mean ± SD		168.3 ± 10.8
Gender, *n* (%)	Female	152 (55.9)
	Male	120 (44.1)
BMI categories, *n* (%)	Underweight	42 (15.4)
	Normal weight	171 (62.9)
	Overweight	44 (16.2)
	Obese	15 (5.5)

**Table 2:** Comparison of subjective well-being and nutrition–exercise behaviors by gender.

Variable	Male Mean ± SD	Female Mean ± SD	Z	*p*	r	FDR-Adjusted *p*
School satisfaction	31.5 ± 9.5	31.1 ± 8.98	−0.41	0.68	0.02	0.68
School-related emotions	7.9 ± 2.5	7.53 ± 2.65	−1.03	0.30	0.06	0.60
Psychological/Dependent Eating	31.59 ± 8.36	29.98 ± 6.61	−1.63	0.10	0.10	0.60
Healthy Nutrition–Exercise	42.92 ± 10.29	42.05 ± 8.48	−0.80	0.42	0.05	0.63
Unhealthy Nutrition–Exercise	41.68 ± 9.57	42.16 ± 7.56	−0.49	0.62	0.03	0.68
Meal Regularity	17.34 ± 6.55	16.33 ± 5.47	−1.29	0.20	0.08	0.60

Mann–Whitney U test was used. r values indicate effect sizes. Benjamini–Hochberg FDR-adjusted *p*-values are reported for transparency.

**Table 3:** Comparison of subjective well-being and nutrition–exercise behaviors across BMI categories.

Variable	Underweight Mean ± SD	Normal Weight Mean ± SD	Overweight Mean ± SD	Obese Mean ± SD	H (χ^2^)	*p*	FDR- Adjusted *p*	ε^2^
School satisfaction	30.38 ± 8.24	32.11 ± 9.37	30.00 ± 9.78	28.46 ± 8.95	3.95	0.26	0.52	0.004
School-related emotions	7.38 ± 2.28	7.81 ± 2.45	8.04 ± 2.75	7.26 ± 3.61	2.65	0.44	0.53	0.000
Psychological/Dependent Eating	29.54 ± 7.45	31.49 ± 7.86	30.26 ± 7.47	29.33 ± 6.21	2.67	0.44	0.53	0.000
Healthy Nutrition–Exercise	40.42 ± 7.73	43.25 ± 10.15	42.09 ± 9.20	41.60 ± 6.96	4.11	0.25	0.52	0.004
Unhealthy Nutrition–Exercise	39.71 ± 7.29	42.15 ± 9.33	41.84 ± 8.13	45.20 ± 5.74	6.51	0.09	0.52	0.013
Meal Regularity	17.35 ± 6.03	16.90 ± 6.05	16.72 ± 6.31	16.00 ± 6.93	0.46	0.93	0.93	0.000

Kruskal–Wallis H test was used. Effect size values are reported as epsilon-squared (ε^2^).

**Table 4:** Correlation matrix of study variables.

Variable	1	2	3	4	5	6
1. Psychological/Dependent Eating	1.00	0.45 **	0.47 **	0.54 **	0.00	−0.01
2. Healthy Nutrition–Exercise Behavior	0.45 **	1.00	0.62 **	0.25 **	0.27 **	0.19 **
3. Unhealthy Nutrition–Exercise Behavior	0.47 **	0.62 **	1.00	0.34 **	0.12 *	0.15 *
4. Meal Regularity	0.54 **	0.25 **	0.34 **	1.00	−0.02	−0.01
5. School Satisfaction	0.00	0.27 **	0.12 *	−0.02	1.00	0.48 **
6. School-related Emotions	−0.01	0.19 **	0.15 *	−0.01	0.48 **	1.00

Spearman rho correlation coefficients are presented. * *p* < 0.05, ** *p* < 0.01.

**Table 5:** Multiple regression analysis examining associations with subjective well-being at school.

Variables	B	Std. Error	β	t	*p*	95% CI
Constant	27.716	6.026	—	4.599	<0.001	15.85, 39.58
Age	0.166	0.310	0.032	0.536	0.592	−0.44, 0.78
BMI	−0.004	0.067	−0.037	−0.626	0.532	−0.17, 0.09
Psychological/Dependent Eating	−0.239	0.118	−0.171	−2.030	0.043	−0.47, −0.01
Healthy Nutrition–Exercise	0.527	0.101	0.380	4.227	<0.001	0.23, 0.63
Unhealthy Nutrition–Exercise	−0.022	0.114	−0.018	−0.190	0.850	−0.25, 0.20
Meal Regularity	−0.014	0.126	−0.008	−0.113	0.910	−0.26, 0.23

Dependent variable: Subjective well-being at school.

## Data Availability

The datasets generated and/or analyzed during the current study are not publicly available because they contain data collected from adolescent participants, and public sharing could compromise participant confidentiality. The datasets are available from the corresponding author on reasonable request, subject to ethical approval and institutional privacy regulations.

## Abbreviations

The following abbreviations are used in this manuscript:

BMI	Body Mass Index
